# The Bovine Antimicrobial Peptide Lactoferricin Interacts with Polysialic Acid without Loss of Its Antimicrobial Activity against *Escherichia coli*

**DOI:** 10.3390/ani10010001

**Published:** 2019-12-18

**Authors:** Andrea Kühnle, Christina E. Galuska, Kristina Zlatina, Sebastian P. Galuska

**Affiliations:** 1 Institute of Reproductive Biology, Leibniz Institute for Farm Animal Biology (FBN), Wilhelm-Stahl-Allee 2, 18196 Dummerstorf, Germany; kuehnle@fbn-dummerstorf.de (A.K.); Galuska.christina@fbn-dummerstorf.de (C.E.G.); Zlatina@fbn-dummerstorf.de (K.Z.); 2 Institute of Biochemistry, Faculty of Medicine, Justus-Liebig-University, Friedrichstr. 24, 35392 Giessen, Germany

**Keywords:** lactoferrin, lactoferricin, polysialic acid, sialic acids, antimicrobial peptides, innate immune system

## Abstract

**Simple Summary:**

Bovine milk contains a high concentration of the protein lactoferrin. It is an important antimicrobial biomolecule, which is also present in other bodily fluids like blood and semen. However, not only the intact protein but also its cleavage products have antimicrobial activity. Perhaps, the best-known cleavage product of lactoferrin is the peptide lactoferricin that has significant antimicrobial capacity against a broad range of pathogens such as enterohemorrhagic *Escherichia coli* (EHEC). Interestingly, lactoferricin can interact with the sugar polymer polysialic acid, which is also present in milk, blood, and semen. In the present study, we tested if the binding to polysialic acid influences the biological activity of bovine lactoferricin. Remarkably, neither different amounts of polysialic acid nor different chain lengths of this sugar polymer influenced the antimicrobial activity of lactoferricin. The ability of polysialic acid to bind and not inactivate lactoferricin may allow the development of novel endogenous and biodegradable polysialylated surfaces and/or hydrogels, which can be loaded with the antimicrobial peptide lactoferricin for biomedical applications in veterinary and human medicine.

**Abstract:**

The lactoferrin-derived peptide lactoferricin (LFcin) belongs to the family of antimicrobial peptides, and its bovine form has already been successfully applied to counteract enterohemorrhagic *Escherichia coli* (EHEC) infection. Recently, it was described that LFcin interacts with the sugar polymer polysialic acid (polySia) and that the binding of lactoferrin to polySia is mediated by LFcin, included in the N-terminal domain of lactoferrin. For this reason, the impact of polySia on the antimicrobial activity of bovine LFcin was investigated. Initially, the interaction of LFcin was characterized in more detail by native agarose gel electrophoresis, demonstrating that a chain length of 10 sialic acid residues was necessary to bind LFcin, whereas approximately twice-as-long chains were needed to detect binding of lactoferrin. Remarkably, the binding of polySia showed, independently of the chain length, no impact on the antimicrobial effects of LFcin. Thus, LFcin binds polySia without loss of its protective activity as an antimicrobial peptide.

## 1. Introduction

Lactoferrin is a multifunctional iron-binding glycoprotein with strong ability to modulate and support the immune system [1,2,3,4,5,6,7]. Interestingly, lactoferrin can be found in nearly all bodily fluids. Lactoferrin can be excreted by epithelial cells, and the highest amounts have been determined in breast milk [8,9]. Besides epithelial cells, neutrophil granulocytes belong to the major producer of lactoferrin and, thus, high concentrations of lactoferrin can be found at inflammatory hotspots [7,8,10].

Recently, the linear carbohydrate polysialic acid (polySia) has been identified as a probable binding partner for lactoferrin [11,12]. The building units of this polysaccharide are sialic acids, a group of α-keto acids with a nine-carbon backbone [13]. The sialic acid residues are mainly attached to each other via α2,9 and/or α2,8 linkages [14]. In mammals, however, only α2,8-linked polymers, consisting of N-acetylneuraminic acid (Neu5Ac) residues, seem to be generated, modulating numerous physiological processes, such as the development of the brain and other organs, in addition to immunological mechanisms [13,15,16,17,18,19,20,21,22]. Interestingly, polySia and lactoferrin can be detected in the same bodily fluids, such as ejaculates, blood, and milk, suggesting that polySia influences the functional properties of lactoferrin [1,12,23,24,25]. Indeed, polySia has been shown to support the activity of lactoferrin in inhibiting the release of neutrophil extracellular traps (NET) [3,12]. NET is a pathogen-catching meshwork of decondensed DNA and antimicrobial molecules that can be formed by neutrophil granulocytes during inflammation [26], and polySia is only one example of several carbohydrate-based mechanisms that influence the formation and functionality of NET [27]. Besides the interplay with lactoferrin during NET formation, polySia seems to modulate the binding of lactoferrin to DNA of already-released NET filaments [11]. This might be possible, since both DNA and polySia molecules mainly interact with the N-terminal domain of lactoferrin [11,28]. This domain is one of the main structural differences between lactoferrin and its iron-transporting family member, transferrin. Interestingly, this cationic domain can be released under acidic conditions or by proteolytic cleavage with pepsin. The resulting peptide is called lactoferricin (LFcin), and besides mammalian proteases, bacterial enzymes are also able to initiate the release of LFcin. The formation of LFcin and other antimicrobial degradation products of lactoferrin was observed in the stomach and other areas during inflammation [6,29,30,31]. The proteolytic release might be an advantage due to the commonly stronger antimicrobial activity of LFcin compared to lactoferrin [5,6,29,32,33,34]. These antibacterial effects, in addition to the observation that bacteria show increasing resistance against antibiotics, make LFcin an interesting alternative or additional therapeutic agent in veterinary and human medicine [34].

It has already been shown that polySia interacts with the LFcin-containing domain in lactoferrin, as well as with the released antimicrobial peptide LFcin [11,12]. Remarkably, polySia also interacts with histones [35,36], which are, like LFcin, denoted as antimicrobial peptides [37,38,39]. Besides the best known function of histones in the packaging of DNA, extracellular histones and their fragments are part of the innate immune system [40], and their antimicrobial capacities have been described in many animal classes, such as insects, fish, amphibians, birds, and mammals [41,42,43,44,45]. In the case of histones, it is known that polySia influences their interactions and/or activity in a chain-length-dependent manner [46,47,48]. However, this interaction does not necessarily have an effect on the functionality of the interaction partners. For instance, the antimicrobial activity of histone H3 is inhibited by polySia, whereas those of the lysine-rich histone H1 is not affected in the presence of polySia [49]. For this reason, we wanted to investigate if polySia has an impact on the antimicrobial capacity of LFcin. To this end, we first characterized the interaction of LFcin and polySia in more detail by using native gel electrophoresis and ELISA with defined chain lengths of polySia. Furthermore, the impact of LFcin on bacterial growth was investigated in the presence of polySia.

## 2. Materials and Methods

### 2.1. Materials

The reagents used for all the following experiments were of analytical grade. The *Escherichia coli* strain BL21 (DE3) was kindly provided by the lab of Joachim Weitzel. In the experiments, lactoferrin from bovine milk (Sigma-Aldrich, Steinheim, Germany), bovine LFcin (B25; Bachem, Bubendorf, Switzerland), Neu5Ac (MonoSia; Carbosynth, Compton, UK), and colominic acid (polySia) (Gerbu, Heidelberg, Germany) were used. Lipopolysaccharides (LPS) were removed from polySia with C18 cartridges (ThermoFisher Scientific, Dreieich, Germany), as described in the manufacturer’s manual.

### 2.2. Fractionation and Analysis of Neu5Ac Polymers

In order to obtain polySia fractions with defined degrees of polymerization (DP), commercially available polySia (a heterogeneous mixture of different chain lengths) was separated by anion-exchange chromatography [46,47]. To receive greater amounts of shorter polySia chain lengths (for native agarose gel electrophoresis and competitive ELISA), polySia was previously partially hydrolyzed. Therefore, polySia was incubated in a reaction buffer (9 mM sodium hydrosulfite, 0.5 M β-mercaptoethanol, 20 mM trifluoroacetic acid [TFA]) for 45 min at 55 °C. To stop the hydrolysis, 20% 1 M NaOH (*v*/*v*) was added. PolySia separation took place with MilliQ water (E1) and 2 M ammonium acetate buffer (E2) on a DNAPac™ PA100 column (22 mm × 250 mm, 13 μm; ThermoFisher Scientific) at a flow rate of 2.5 mL/min. The gradient was the following: 0 min = 0% (*v*/*v*) E2, 5 min = 0% (*v*/*v*) E2, 20 min = 26% (*v*/*v*) E2, 30 min = 34% (*v*/*v*) E2, 45 min = 38% (*v*/*v*) E2, 85 min = 44% (*v*/*v*) E2, 110 min = 100% (*v*/*v*) E2, 140 min = 100% (*v*/*v*) E2, 141 min = 0% (*v*/*v*) E2. To remove ammonium acetate, the samples were dried by lyophilization and evaporation.

Then, 0.01% of the gained fractions were quantified with the eluents methanol/acetonitrile/water/ trifluoroacetic acid (TFA) (4:4:92:0.1; E3) and methanol/acetonitrile/water/TFA (45:45:10:0.1; E4) on a Superspher 100 C-18 column (250 mm × 40 mm; Merck-Hitachi, Darmstadt, Germany). After hydrolysis in 0.2 M TFA for 4 h at 80 °C, the samples were dried, and Neu5Ac residues were labeled with 4,5-methylene dioxybenzene (DMB) in 80 µL DMB reaction buffer (9 mM sodium hydrosulfite, 0.5 M β-mercaptoethanol, 20 mM TFA) for 2 h at 55 °C; the reaction was stopped with 20 µL 0.2 M NaOH (*v*/*v*) [50,51]. The used gradient was the following: 0 min = 0% (*v*/*v*) E4, 2 min = 0% (*v*/*v*) E4, 25 min = 5% (*v*/*v*) E4, 31 min = 100% (*v*/*v*) E4, 40 min = 100% (*v*/*v*) E4, 41 min = 0% (*v*/*v*) E4, 55 min = 100% (*v*/*v*) E4. The flow rate was 0.25 mL/min.

To control the separated chain lengths, 100 ng of each fraction was mildly labeled with DMB in 80 µL DMB reaction buffer (overnight at 11 °C), and 20 ng were analyzed with a DNAPac™ PA100 column (4 mm × 250 mm, 13 μm; ThermoFisher Scientific) [52]. The used gradient was the following: 0 min = 0% (*v*/*v*) E2, 5 min = 0% (*v*/*v*) E2, 15 min = 5% (*v*/*v*) E2, 30 min = 21% (*v*/*v*) E2, 55 min = 33% (*v*/*v*) E2, 100 min = 43% (*v*/*v*) E2, 101 min = 100% (*v*/*v*) E2, 110 min = 100% (*v*/*v*) E2, 111 min = 100% (*v*/*v*) E2. The flow rate was 1 mL/min. PolySia digested with active endoneuraminidase (EndoN, 0.1 µg) was analyzed under the same conditions.

### 2.3. Electrophorese on Native Agarose Gel

For the interaction analysis, lactoferrin and LFcin were separated by native gel electrophoresis in the absence and presence of Neu5Ac and different chain length of polySia, as previously described in detail [12,53]. To this end, the samples were loaded on an agarose gel (2%, *w*/*v*) (lactoferrin and LFcin, 10 µg/lane; Neu5Ac and sialic acid polymers, 5 µg/lane) and separated for 5 h at 80 V (running buffer: 25 mM Tris/HCl, 19.2 mM glycine, pH 8.5). Afterwards, the proteins were fixed in 45% methanol/7.5% acetic acid (*v*/*v*) overnight and colored with Coomassie blue (Roti-Blue, Carl-Roth), and the gel was de-stained with 25% methanol.

### 2.4. ELISA

ELISA plates were coated with 20 µg/mL of lactoferrin (coating buffer, 15 mM Na_2_CO_3_, 35 mM NaHCO_3_, pH 9.6) for 2 h, followed by two washing steps. Afterwards, Neu5Ac and polySia fractions (160 μg/mL in PBS) were added, and incubation was carried out for 2 h. In a further step, murine anti-LFcin antibody (0.5 μg/mL) was applied to bind unoccupied LFcin binding sides. For a detailed description, please see [12].

### 2.5. Bacterial Growth Assay

Bacterial growth was analyzed with the Bacteria Counting Colorimetric Assay Kit (BioVision, Milpitas, CA, USA), which was also used to characterize the impact of histones on *E. coli* [49]. During all the following steps, *E. coli* was cultured at 37 °C under shaking. To generate a preculture, LB medium (1% NaCl [*w*/*v*], 1% peptone [*w*/*v*], 0.5% yeast extract [*w*/*v*]) was inoculated with a frozen *E. coli* stock and incubated overnight. With this preculture, a main culture was inoculated and grown until an OD (600 nm) of 0.29−0.32 was reached. For the bacterial growth experiments, 50 µL of LB medium was added to a 96-well plate. In addition, LB medium containing LFcin (200 µg/mL) and/or polySia (400, 200, or 100 µg/mL), Neu5Ac (400 µg/mL), fractionated polySia (400 µg/mL), or enzymatically cleaved polySia (400 µg/mL) was applied. For the enzymatic digestion of the polymers, polySia (6 mg/mL) was treated with endo N (6.7 μg/mL, 3 h, 37 °C). To the 50 µL of differently modified LB media, 40 µL of bacteria solution (~2.4 × 10^8^ bacteria/mL) and 10 µL of WST/ECS solution (reagents of the Bacteria Counting Colorimetric Assay Kit) were added. Thus, the final concentration of LFcin is 100 µg/mL. The bacterial growth was measured for 150 min in 30 min intervals, at a wavelength of 450 nm.

### 2.6. Statistical Analysis

The calculated values were analyzed with Graph Pad Prism 8.2.1 software using ANOVA and a multiple-comparison Turkey test. Differences were considered statistically significant at *p* < 0.05. Statistically significant differences are indicated: ns, not significant; * *p* < 0.05; ** *p* < 0.01; *** *p* < 0.001; **** *p* < 0.0001.

## 3. Results and Discussion

### 3.1. A Lower DP of PolySia Is Sufficient to Mediate the Binding to LFcin in Comparison to Lactoferrin

Antimicrobial peptides act together as a functional complex to attack the bacterial membrane [54]. If a switch of several LFcin molecules from one polySia chain to the bacterial membrane is possible, it is conceivable that such an accumulation of several LFcin molecules on a polySia chain supports the cooperation of the peptides in the formation of damaging complexes. The LFcin peptides would be directly located in a functional neighborhood.

**Figure 1:**
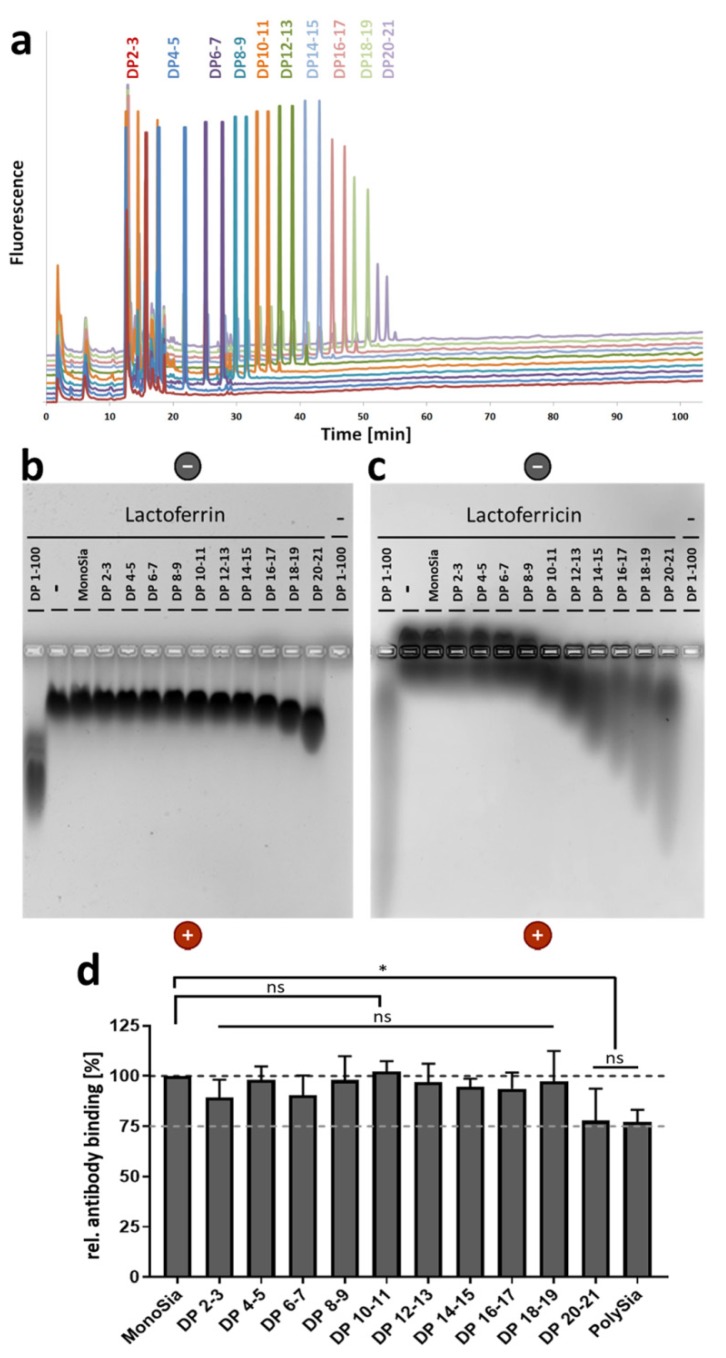
In comparison to lactoferricin (LFcin), higher degrees of polymerization (DPs) are needed for the interaction with lactoferrin. (**a**) The chain lengths of fractionated polysialic acid (polySia) were analyzed using “mild” dioxybenzene (DMB) labeling and anion-exchange chromatography. (**b**) Lactoferrin and (**c**) LFcin were incubated with polySia with chains of defined lengths, and the different samples were analyzed by native gel electrophoresis. In addition, unfractionated polySia (~DP 1–100) was used. Proteins were visualized by staining with Coomassie Blue. To exclude staining of polySia by Coomassie Blue, unfractionated polySia was separated without lactoferrin and LFcin. The gel was loaded with 10 µg/lane of lactoferrin or LFcin and/or 5 µg/mL of carbohydrate. (**d**) A competitive ELISA with an anti-LFcin antibody was performed by using sialic acid with chains of different lengths. Neu5Ac was used as a negative control and set to 100%. Mean values (*n* = 3) and standard deviations are displayed in the diagram. Statistically significant differences are indicated: nonsignificant (ns) and * *p* < 0.05.

To calculate the loading capacity of a polySia chain, it is important to determine the precise number of linked sialic acid residues which are needed to initiate the interaction. In polySia, the degree of polymerization necessary for the interaction with human lactoferrin or bovine LFcin was previously narrowed down to fractions consisting of polymers with a DP between 15 and 24 sialic acid residues [11,12]. Fractions with shorter chains, consisting of 2 up to 14 linked Neu5Ac residues, showed no reliable interaction with lactoferrin and LFcin in these studies. For the present experiments, groups containing only two main chain lengths (DP 2–3, 4–5, 6–7, 8–9, 10–11, 12–13, 14–15, 16–17, 18–19, and 20–21) were collected. In the collected fractions, the chain lengths were tested by anion-exchange chromatography after fluorescent labeling with DMB (Figure 1a). It was not necessary to collect monoSia, since Neu5Ac is commercially available.

To analyze the ability of polySia to interact with lactoferrin, native agarose gels were loaded with LFcin and/or sialic acid chains of different lengths. In addition, lactoferrin was applied by using an identical experimental setup. Thereby, an interaction could be visualized by a migration shift of the peptide/protein to the positive pole. This migration shift might be caused by the polyanionic charge of polySia during the attachment to its binding partners. In the case of lactoferrin, a first weak interaction may start with a DP of 18–19 (Figure 1b). The fraction DP 20–21 significantly influences the migration of lactoferrin. In contrast, already shorter chains influence the migration of LFcin. Here, a detectable interaction started with DP 10–11 (Figure 1c).

Since lactoferrin is much bulkier than LFcin, it is likely that an interaction with short polySia chains may only slightly influence the migration in the gel. Thus, an interaction with short chains might be overlooked. In order to confirm the obtained results, lactoferrin was coated on ELISA plates. Thereafter, polySia fractions according to their DP were added. Subsequently, an antibody against the LFcin-containing domain of lactoferrin was added. The antibody could easily bind the unoccupied N-terminal domain, whereas polySia inhibited the binding [12]. In line with the results from the native agarose gel, polySia with DP 20–21 and unfractionated polySia were able to inhibit the antibody binding (Figure 1d). Thus, in comparison with LFcin, twice-as-long polymers were needed to mediate an interaction.

The differences between LFcin and lactoferrin regarding the required chain length for polySia binding might be the results of conformational changes of LFcin after proteolytic release. It is known that bovine LFcin can change its conformation from an α-helical structure to a twisted β-sheet. This transformation might be also the reason for a better binding of LFcin to bacterial membranes and the stronger antibacterial effect of LFcin in comparison to lactoferrin [5,6,55]. In addition, in lactoferrin, large molecular surface areas of LFcin are hidden by other domains of the protein. Furthermore, it needs to be mentioned that molecular dynamic simulation suggested a second binding domain in lactoferrin for the terminal end of a polySia chain [11]. The interaction with two different protein domains is probably needed to stabilize the interaction in the case of lactoferrin.

### 3.2. PolySia Has No Impact on the Antimicrobial Activity of LFcin

**Figure 2:**
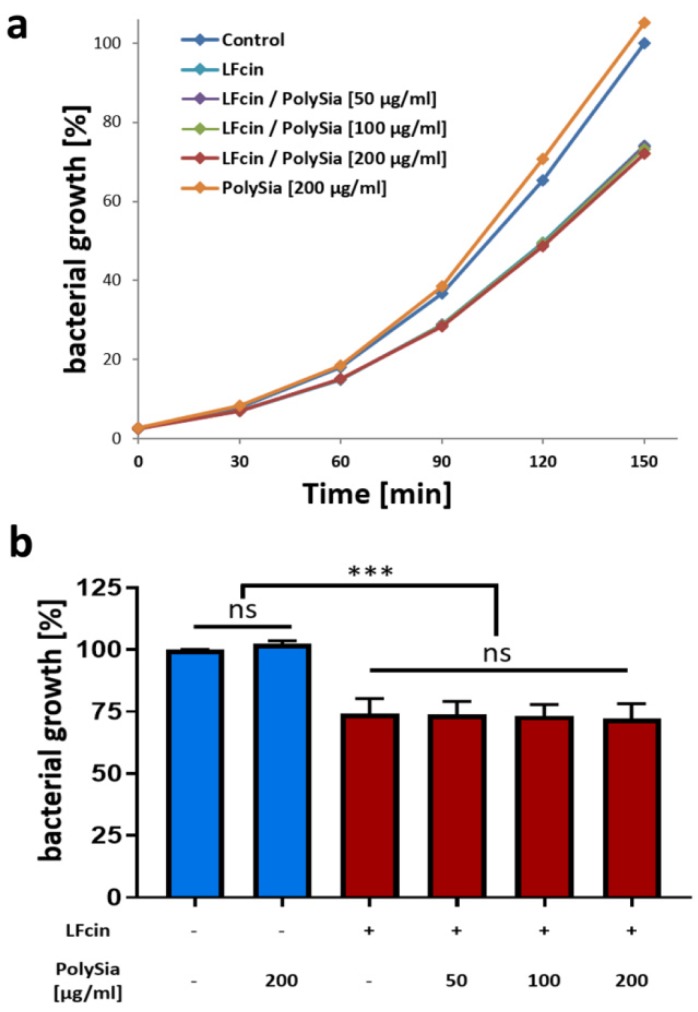
PolySia has no capability to inhibit bacterial growth or the antimicrobial activity of LFcin. (**a**) Bacteria growth curves were obtained in the absence and presence of LFcin (100 µg/mL) and different amounts of polySia, as indicated in the diagram. In (**a**), growth curves of an exemplary experiment are depicted. Values at 150 min are displayed separately in (**b**). The values for untreated bacteria were set to 100%. Mean values (*n* = 3) and standard deviations are displayed in the diagram. Statistically significant differences are indicated: nonsignificant (ns) and *** *p* < 0.001.

To prove the ability of polySia to influence the antibacterial properties of LFcin, both molecules were tested for their ability to inhibit the growth of bacteria. In bacterial growth assays, LFcin and polySia were separately tested, in addition to a combination of LFcin and different concentrations of polySia. As expected, bacterial growth was inhibited by LFcin (Figure 2a,b). In contrast, polySia had no statistically significant impact on bacterial growth (Figure 2b). Surprisingly, when LFcin was applied together with polySia, its antimicrobial activity was not influenced by polySia. The growth curves in the presence or absence of polySia were very similar, and no statistical differences were observed after 150 min.

**Figure 3:**
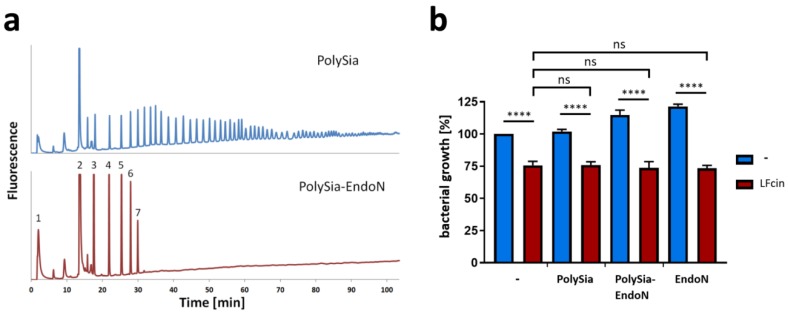
Bacterial growth inhibition by LFcin is not influenced by short sialic acid oligomers. (**a**) PolySia and polySia degraded by endoN were analyzed by anion-exchange chromatography after “mild” DMB labeling. (**b**) Bacterial growth values at 150 min are displayed using polySia (200 µg/mL) or enzymatically degraded polySia (200 µg/mL) in the presence or absence of LFcin (100 µg/mL). Untreated cells were set to 100%. Red bars show LFcin-treated samples, whereas blue bars represent data without LFcin treatment. Mean values (*n* = 3) and standard deviations are displayed in the diagram. Statistically significant differences are indicated: nonsignificant (ns) and **** *p* < 0.0001.

In order to test weather short or long chains of polySia may have an impact on LFcin’s antimicrobial effect, endoN was used to cut the polySia mixture [56]. EndoN works very well because it degrades polySia to mainly short oligomers with a DP < 8 [57]. The digestion was controlled by anion-exchange chromatography after DMB labeling. The obtained chromatograms demonstrated that the degradation was sufficient (Figure 3a). However, also the resulting fragments of polySia exhibited no ability to influence the effects of LFcin (Figure 3b). As described above, a DP higher than 9 was needed to visualize an impact on the migration of LFcin (Figure 1c). Thus, the obtained degradation products (DP < 8) might be too short.

**Figure 4:**
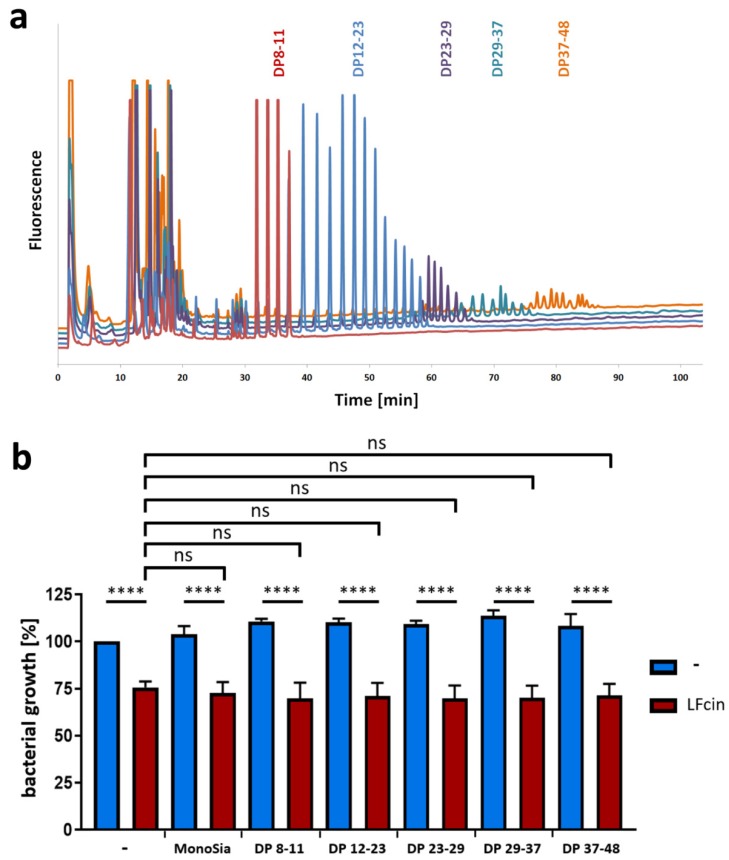
Bacterial growth inhibition by LFcin is not influenced by different chain lengths of polySia. (**a**) Fractionated polySia samples with different degrees of polymerization (DP 8–48) were analyzed by using “mild” DMB labeling and anion-exchange chromatography. (**b**) Bacterial growth values after 150 min are displayed after treatment with LFcin (100 µg/mL) and defined polySia fractions (200 µg/mL). Control (without treatment) was set to 100%. Red bars show LFcin-treated sample datasets, and blue bars show sample datasets without LFcin treatment. Mean values (*n* = 3) and standard deviations are displayed in the diagram. Statistically significant differences are indicated: nonsignificant (ns) and **** *p* < 0.0001.

For this reason, five groups of polySia with chain lengths between 8 and 48 were collected, and the DP was analyzed by HPLC (Figure 4a). On the basis of the results of native gel electrophoresis, the chains have the theoretical capacity to bind one to four LFcin molecules. These fractions were used in combination with LFcin for the bacterial growth assays. The results in Figure 4b clearly depict the capacity of LFcin to inhibit bacterial growth despite the presence of polySia fractions of rising DPs. Thus, polySia can bind LFcin, but it has no impact on its antimicrobial activity. Comparable results were also obtained for histones H1 and H2A, as previously described [49].

## 4. Conclusions

Interestingly, it was shown that polySia and lactoferrin can interact in several bodily fluids, that polySia supports lactoferrin to inhibit the release of NET, and that polySia influences the binding of lactoferrin to already exposed NET fibers. Since polySia also interacts with the lactoferrin-derived peptide LFcin, an effect of polySia on the biological function of the antimicrobial peptide was also conceivable. Since the binding of lactoferrin is initiated by its LFcin-containing domain, a modulation of LFcin binding to NET or to polysialylated immune cells, such as dendritic cells, is likely [58].

Recently, a therapeutic effect of bovine LFcin after oral intake was reported for the prevention of enterohemorrhagic *E. coli* (EHEC) infection in a mouse model [59], demonstrating, like numerous other studies, the potential of this antimicrobial peptide in veterinary and human medicine (reviewed in [34]). However, neither different amounts of polySia nor different polySia DPs can influence this ability of LFcin. Particularly, the ability to bind and not inactivate LFcin might be a big advantage, if polysialylated surfaces can be loaded with LFcin. One additional point to note is that lactoferrin, LFcin, and polySia are endogenous biomolecules and biodegradable. For this reason, polySia and LFcin might be a powerful combination to develop novel therapeutic strategies, such as polysialylated surfaces and/or hydrogels which can be equipped with the detachable antimicrobial peptide LFcin.
